# Transcultural validation and factorial invariance assessment of the EEA Resilience Scale in health sciences students from eight Latin American countries

**DOI:** 10.1186/s41155-026-00387-0

**Published:** 2026-03-26

**Authors:** José Gamarra-Moncayo, Estela Marcelo-Ascencio, Lindsey W. Vilca, Carolina More-Toro, Sara Huerta-González, Rubén Eduardo Vázquez-García, Luz Marina Alonso Palacio, Wilson Pastén Hidalgo, Anyel Bertel De la Hoz, Aleyda I. Parra-Castillo, Eugenia S. González-Díaz, Nuvia Estrada-Méndez, Luis German Montero Saldaña, Sabrina A Carranza, María Laura Frutos, Claudia Arispe-Alburqueque, Javiera Santana-Abásolo, Margarett Cuello-Pérez, J.E. Rod, Mildred Amparo Sandoval, Lesbia Tirado-Amador, Gary Caballero García, Diana Escobar Ospino, Olga Tatiana Jaimes Prada, Andrés Llanos Redondo, Victor P. Díaz-Narváez

**Affiliations:** 1 Facultad de Medicina, Universidad Católica Santo Toribio de Mogrovejo, Chiclayo, Peru; 2 Department of Psychology, Universidad Señor de Sipán, Chiclayo, Peru; 3 Facultad de Ciencias de la Salud, Universidad de Atacama, Copiapó, Chile; 4 Faculty of Nursing, Universidad Veracruzana, Poza-Tuxpan, Mexico; 5 División de Ciencias de la Salud, Universidad del Norte, Barranquilla, Colombia; 6 Faculty of Medicine, Universidad del Sinú, Cartagena, Colombia; 7 Facultad de Ciencias de la Salud, Universidad del Atlántico, Barranquilla, Colombia; 8 Faculty of Medicine, Universidad Central del Este, San Pedro de Macorís, Dominican Republic; 9 Vicerrectoría de Investigaciones, Universidad Evangélica de El Salvador, San Salvador, El Salvador; 10 Facultad de Ciencias Naturales y Exactas., Universidad Autónoma de Chiriquí, Chiriquí, Panama; 11 Facultad de Jurisprudencia, Universidad de Cuenca, Cuenca, Ecuador; 12 Facultad de Ciencias de la Salud, Universidad Católica de Córdoba, Córdoba, Argentina; 13 Facultad de Ciencias de la Salud, Universidad Nacional Federico Villarreal, Lima, Peru; 14 Dirección de Docencia, Universidad de Concepción, Concepción, Chile; 15 Escuela de Ciencias de la Salud, Universidad Nacional Abierta y a Distancia, Cartagena, Colombia; 16 Facultad de Ciencias de la Salud, Universidad Autónoma de Santa Ana, Santa Ana, El Salvador; 17 Facultad de Ciencias de la Salud, Universidad del Magdalena, Santa Marta, Colombia; 18 Facultad de Ciencias de la Salud, Universidad de Pamplona, Pamplona, Colombia; 19 Facultad de Odontología, Universidad Andres Bello, Santiago, Chile

**Keywords:** Resilience, Psychometric properties, Health sciences, University students, Latin America, Transcultural validity

## Abstract

**Background:**

Resilience is understood as a person’s ability to adapt positively in the face of adversity, overcome difficult situations, and, in many cases, emerge stronger from them. The objective of this study was to conduct a transcultural validation of the Engineering, Ecological and Adaptive Resilience Scale in health sciences students from eight Latin American countries.

**Methods:**

A total of 18,528 students participated in instrumental cross-sectional design. Internal structure was assessed through confirmatory factor analysis, reliability was estimated using the omega coefficient, factorial invariance by sex and country was examined, sex differences were analyzed, and percentile norms were established.

**Results:**

The three-dimensional structure of the EEA Resilience Scale showed adequate fit indices in all countries and in the total sample, although Ecuador, Panama, and Peru exhibited Root Mean Square Error of Approximation values slightly above expected thresholds. Reliability was satisfactory, except in El Salvador. Strict invariance by sex and country was confirmed. Country and sex differences showed trivial effect sizes. Percentile-based norms were proposed at five levels.

**Conclusions:**

Overall, the findings indicate that the EEA Resilience Scale is a valid, reliable, and transculturally robust measure for assessing resilience. The scope of the study is discussed.

## Introduction

Resilience is an essential psychological resource that enables individuals to confront, adapt to, and recover from adverse situations, playing a key role in well-being and optimal functioning (Elyasi et al., 2024). Its importance is particularly evident among health sciences students, who undergo highly demanding training processes characterized by intense academic workloads, continuous emotional exposure, and high levels of stress (Azim et al., 2025; Bakhtiar et al., 2025; Byrne et al., 2025; Song et al., 2023). The literature reports significant prevalence rates of anxiety, depression, and burnout, with international studies documenting up to 27% for depressive symptoms and 90% for stress (Rotenstein et al., 2016; Lei et al., 2025; Otaki et al., 2025; Mohammed et al., 2024; Zila-Velasque et al., 2024). Resilience—understood as the capacity to generate positive psychophysiological outcomes when facing adversity (Ammar et al., 2023; Azim et al., 2025; Bakhtiar et al., 2025)—has been associated with lower levels of anxiety and depression, as well as with better overall mental health (Song et al., 2023).

Resilience is not a static trait; rather, it is modulated by variables such as sex, age, culture, and worldview (Elyasi et al., 2024; Fletcher & Sarkar, 2013; Mohammed et al., 2024; Wongpakaran et al., 2023). Although some studies in the health sciences have found that men exhibit higher resilience levels (Ammar et al., 2023; Mohammed et al., 2024), other research reports no differences (Al Omari et al., 2023; Chow et al., 2018) or even higher levels in women (Sull et al., 2015). Likewise, cross-cultural variability in the expression of the construct has been documented (Otaki et al., 2025; Mohammed et al., 2024). This heterogeneity has driven the development of more integrative models such as the Engineering, Ecological and Adaptive (EEA) Resilience Model, which emerged from the analysis of five widely used scales and is grounded in Holling’s ecological systems theory (Maltby et al., 2015, 2016, 2017; Maltby & Hall, 2022).

The EEA model identifies three facets: engineering resilience, referring to the capacity to bounce back quickly; ecological resilience, linked to the ability to absorb disruptions; and adaptive capacity, related to integrating new processes in response to change (Maltby et al., 2015, 2016). These dimensions have demonstrated significant associations with personality traits, coping styles, and psychological well-being (Maltby et al., 2016). Although multiple instruments exist to assess resilience—such as the CD-RISC (Bakhtiar et al., 2025; Ammar et al., 2023; Park et al., 2023), the Wagnild and Young Scale (1993), the Dispositional Resilience Scale (Bartone, 2013), the Brief Resilience Scale (Smith et al., 2008), and the RI-9 (Wongpakaran et al., 2023)—there remains a need for measures with clear and replicable structures (Huerzeler et al., 2025). In addition, scholars have recommended complementing factorial research with person-centered approaches such as LPA or LCA (Song et al., 2023).

In the Latin American context, some studies have evaluated the psychometric properties of resilience scales, particularly the Connor–Davidson Resilience Scale and its abbreviated versions. For example, research conducted in Peru has reported adequate reliability and factorial validity for its abbreviated versions of 10 and 7 items in unidimensional models (Bernaola et al., 2022; Domínguez-Lara et al., 2019; Seperak-Viera et al., 2023), although a four-dimensional model was also reported for the 22-item version (Domínguez-Cancino et al., 2022). On the other hand, in Colombian samples (Guarnizo et al., 2019), the unidimensionality of the scale has been replicated. Although these studies provide valuable evidence for the measurement of resilience in Latin America, most instruments operationalize the construct as a single global dimension. In contrast, the Engineering, Ecological, and Adaptive (EEA) model of resilience conceptualizes resilience as a multidimensional system composed of complementary facets, offering a more nuanced profile of how individuals recover from adversity, absorb disturbances, and adapt to changing circumstances (Maltby et al., 2015, 2016). This multidimensional perspective may be particularly useful in demanding educational contexts such as training in the health sciences, where different resilience processes may operate simultaneously.

Although the EEA model’s structure has been replicated in the United States, Japan, and Poland, and even bifactor structures were detected (Maltby et al., 2016); as also mentioned in the previous paragraph, evidence in Latin America is limited and restricted to country-specific studies without comprehensive transcultural evaluations (Acosta-Martínez et al. 2024; Díaz-Narváez et al. 2025a, b, c). Validating this instrument among Latin American students is especially relevant given their elevated exposure to emotional demands, the impact of resilience on academic and professional performance (Sanderson & Brewer, 2017), and its value for designing preventive and psychological support interventions (Azim et al., 2025; Wadi et al., 2024). Additionally, understanding variations by sex and culture enables more contextualized and effective strategies (Ammar et al., 2023; Lei et al., 2025; Jalali et al., 2025).

In this regard, the present research is relevant for multiple reasons. Academically, health sciences students face stress levels and demands that exceed those of other fields, affecting their well-being and performance (Byrne et al., 2025; Elyasi et al., 2024). Thus, resilience is essential not only for their academic and personal success (Sanderson & Brewer, 2017) but also for the development of their professional identity (Jalali et al., 2025). A multidimensional instrument such as the EEA provides a more nuanced understanding of coping strategies than a single global score, which is crucial given the inherent heterogeneity of this construct (Song et al., 2023). Variations in resilience by sex and culture underscore the need for a validated approach that considers regional specificities (Ammar et al., 2023; Lei et al., 2025).

Interventions grounded in an accurate understanding of resilience facets are indispensable for training physicians who are more competent, empathetic, and capable of facing the adversities inherent to their profession (Byrne et al., 2025).

Therefore, this study aimed to conduct a cross-cultural validation of the EEA Resilience Scale in health sciences students from eight Latin American countries, evaluating its internal structure, reliability, invariance by sex and country, differences based on sex and country, and establishing percentile norms.

## Materials and methods

### Design

This study employed a cross-sectional instrumental design focused on the evaluation of psychometric properties (Ato et al., 2013), following recommended guidelines for validation studies within the framework of Classical Test Theory (Muñiz, 2018).

### Participants

The sample consisted of 18,528 health sciences students (61.6% women) from eight Latin American countries (Chile, Colombia, Ecuador, El Salvador, Mexico, Panama, Peru, and the Dominican Republic), ranging in age from 16 to 60 years (M = 21.12, SD = 3.19). A non-probabilistic convenience sampling method was used (Goodwin & Goodwin, 2018). Given the final sample size, factorial stability can be ensured, reducing the risk of non-convergence issues in psychometric analyses (Gamarra-Moncayo & Prada-Chapoñan, 2025).

### Instrument

The Engineering, Ecological and Adaptive (EEA) Resilience Scale assesses three dimensions through 12 items using five response options. Previous studies have reported adequate psychometric properties. It includes three dimensions (engineering, ecological, and adaptive). The confirmatory factor analysis yielded acceptable fit indices: χ²(df) = 98.56(51), CFI = 0.95, NNFI = 0.94, RMSEA = 0.075, and SRMR = 0.06. Reliability, evaluated through Cronbach’s alpha, was satisfactory across dimensions (α > 0.70). Each factor has also been shown to relate differently to personality traits (Big Five model) and coping styles and has predictive capacity for psychological well-being.

For the present study, five response options were used, ranging from strongly disagree (1) to strongly agree (5), as this number of categories typically produces more stable factor estimations (Rhemtulla et al., 2012).

### Procedure

The instrument underwent a translation and back-translation process in each participating country, followed by expert review (Judgment of Experts) by two Full Professors, two Higher Education Pedagogues, and one Social Psychologist. After approval, a pilot test was conducted with 20 randomly selected health sciences students from different academic years to verify item comprehension.

The scale was administered in physical (printed) format, in person, under the supervision of previously trained instructors, and applied within students’ academic environments (classrooms). Participation was voluntary and anonymous. All participants read, accepted, and signed written informed consent; in the case of minors, consent was additionally obtained from parents or legal guardians. Administration procedures were standardized across all participating countries.

Stratification was based on academic year within each participating faculty. This approach allowed the collection of representative samples at each institution and reduced the risk of selection bias.

### Data analysis

Univariate descriptive statistics were calculated for each country and for the total sample. Confirmatory Factor Analysis (CFA) was then conducted using the Weighted Least Squares Mean and Variance Adjusted (WLSMV) estimator, appropriate for ordinal indicators based on the polychoric correlation matrix (Kline, 2023), given the five-category response format.

Model fit was evaluated using the following criteria: Comparative Fit Index (CFI > 0.90), Tucker-Lewis Index (TLI > 0.90), Root Mean Square Error of Approximation (RMSEA < 0.08), and Standardized Root Mean Square Residual (SRMR < 0.08) (Whittaker & Schumacker, 2022).

In order to evaluate the degree of essential unidimensionality of the construct and the appropriateness of interpreting a bifactor model, the additional indices Explained Common Variance (ECV) and Percentage of Uncontaminated Correlations (PUC) were calculated. The ECV index represents the proportion of the common variance explained by the general factor in relation to the specific factors, whereas PUC indicates the proportion of correlations among items that reflect only the influence of the general factor. The literature suggests that high values of ECV (0.60–0.70 or higher) and PUC (0.70 or higher) indicate that the construct can be considered essentially unidimensional and that interpretation of the general factor is appropriate. Furthermore, when PUC ≥ 0.80, even moderate ECV values may be sufficient to support the essential unidimensionality of the instrument (Domínguez-Lara & Rodríguez, 2017).

Measurement invariance by sex and country was tested using thresholds of Delta Comparative Fit Index (ΔCFI ≤ 0.01) and Delta Root Mean Square Error of Approximation (ΔRMSEA ≥ 0.015) (Chen, 2007). Internal consistency was assessed using the omega coefficient, with values above 0.70 considered acceptable (Viladrich et al., 2017). For the bifactor model, both the specific omegas for each dimension and the hierarchical omega for the general dimension were calculated, where values greater than 0.30 and 0.70, respectively, are expected (Domínguez-Lara & Rodríguez, 2017).

After establishing measurement invariance across countries, their differences in resilience scores were examined using a one-way analysis of variance (ANOVA). It was evaluated using omega squared (ω²) as an indicator of effect size, which allowed estimating the proportion of variance in resilience scores attributable to country membership. The magnitude of ω² was interpreted following conventional benchmarks, small (0.01), medium (0.06) and large (0.14) as suggested in the literature (Domínguez-Lara, 2018).

Sex differences were examined using Student’s t-test, which is robust for large samples even when data are skewed (Fagerland, 2012), and effect sizes were calculated using Cohen’s d (Domínguez-Lara, 2018). Percentile-based norms were established following recommended procedures (Muñiz, 2018) and categorized into five levels, consistent with prior EEA studies (Díaz-Narváez et al. 2025a).

Analyses were conducted using JASP v0.95.2 and the R programming language in the RStudio environment, employing the psych v2.5.6, lavaan v0.6-19, and semTools v0.5-7 packages.

### Ethical considerations

The Research Project and Informed Consent were approved by the Institutional Bioethics Committee of Universidad Andrés Bello (UNAB), Santiago, Chile (July 2022), Act No. 020/2022. All participating universities acknowledged and adhered to this institutional approval. The study followed the ethical principles outlined in the Declaration of Helsinki.

Participation was entirely voluntary and anonymous. For participants under 18 years of age, informed consent was obtained from parents or legal guardians, in addition to student assent. All participants agreed to the use of their data for analysis and publication of results.

This study includes data obtained from health sciences students in dentistry, nursing, and medicine previously used by the authors in published research involving the resilience variable (Acosta-Martínez et al. 2024; Díaz-Narváez et al. 2025a, b, c, d, e; Hernández-Álvarez et al., 2025; Méndez-Hurtado et al., 2025). Additionally, this work incorporates unpublished data from students in Kinesiology, Kinesiotherapy, Nutrition, Occupational Therapy, Speech and Language Pathology, Medicine, Dentistry, and Nursing.

## Results

Table 1 shows that, for all countries and for the total sample, the items do not deviate from univariate normality, as their skewness and kurtosis coefficients fall within acceptable ranges (± 2; Bandalos & Finney, 2019).

**Table 1:** Univariate descriptive statistics of the items by country and total sample

Country	Items	M	SD	g1	g2	Items	M	SD	g1	g2
Chile (*n* = 2,385)	1	3.48	1.09	-0.41	-0.50	7	3.88	0.95	-0.73	0.25
	2	3.29	1.09	-0.20	-0.69	8	3.97	0.90	-0.74	0.37
	3	3.32	1.11	-0.21	-0.67	9	3.39	1.15	-0.27	-0.61
	4	3.23	1.12	-0.12	-0.78	10	2.89	1.15	0.07	-0.71
	5	4.27	0.85	-1.16	1.21	11	3.10	1.20	-0.13	-0.84
	6	3.88	0.96	-0.69	0.11	12	3.30	1.25	-0.27	-0.89
Colombia (*n* = 8,345)	1	3.52	1.12	-0.55	-0.35	7	3.86	1.00	-0.76	0.15
	2	3.36	1.13	-0.29	-0.65	8	4.05	0.89	-0.92	0.86
	3	3.47	1.11	-0.39	-0.58	9	3.77	1.05	-0.62	-0.16
	4	3.41	1.14	-0.33	-0.72	10	3.47	1.10	-0.37	-0.50
	5	4.06	0.96	-0.98	0.65	11	3.43	1.13	-0.38	-0.56
	6	3.84	1.02	-0.69	-0.05	12	3.73	1.12	-0.71	-0.12
Ecuador (*n* = 1,822)	1	3.44	1.11	-0.34	-0.58	7	3.69	1.06	-0.58	-0.25
	2	3.26	1.15	-0.08	-0.86	8	3.80	0.99	-0.64	0.00
	3	3.29	1.14	-0.13	-0.86	9	3.57	1.12	-0.55	-0.32
	4	3.21	1.16	-0.06	-0.86	10	3.28	1.17	-0.23	-0.73
	5	3.94	0.96	-0.85	0.50	11	3.44	1.13	-0.41	-0.51
	6	3.68	1.05	-0.55	-0.25	12	3.13	1.29	-0.08	-1.07
El Salvador (*n* = 1,956)	1	3.36	1.22	-0.37	-0.81	7	3.69	1.17	-0.73	-0.25
	2	3.12	1.22	-0.16	-0.91	8	3.80	1.15	-0.90	0.07
	3	3.25	1.25	-0.25	-0.92	9	3.43	1.28	-0.39	-0.88
	4	3.11	1.21	-0.11	-0.92	10	3.11	1.23	-0.10	-0.89
	5	3.97	1.16	-1.09	0.36	11	3.24	1.24	-0.22	-0.90
	6	3.63	1.19	-0.62	-0.48	12	3.48	1.31	-0.48	-0.87
Mexico (*n* = 648)	1	3.37	1.06	-0.29	-0.47	7	3.77	0.99	-0.61	-0.03
	2	3.12	1.07	-0.07	-0.58	8	3.91	0.91	-0.63	0.17
	3	3.23	1.05	-0.07	-0.67	9	3.53	1.12	-0.42	-0.47
	4	3.12	1.07	0.02	-0.74	10	3.07	1.11	-0.01	-0.57
	5	4.12	0.89	-0.93	0.66	11	3.21	1.12	-0.15	-0.63
	6	3.75	1.04	-0.55	-0.25	12	3.59	1.18	-0.51	-0.54
Panama (*n* = 684)	1	3.57	1.00	-0.24	-0.56	7	3.90	0.94	-0.58	-0.09
	2	3.30	0.98	-0.09	-0.35	8	4.11	0.85	-0.60	-0.22
	3	3.42	1.01	-0.10	-0.52	9	3.55	1.03	-0.31	-0.34
	4	3.37	0.99	-0.08	-0.43	10	3.15	1.15	-0.09	-0.62
	5	4.22	0.91	-1.06	0.70	11	3.34	1.15	-0.25	-0.66
	6	3.92	0.94	-0.54	-0.32	12	3.54	1.17	-0.41	-0.63
Peru (*n* = 566)	1	3.46	1.06	-0.32	-0.55	7	3.82	1.01	-0.58	-0.22
	2	3.28	1.09	-0.17	-0.59	8	3.87	0.93	-0.48	-0.27
	3	3.30	1.07	-0.18	-0.64	9	3.23	1.18	-0.11	-0.76
	4	3.26	1.07	-0.16	-0.65	10	2.91	1.22	0.08	-0.89
	5	4.12	0.91	-0.89	0.34	11	3.11	1.21	-0.10	-0.90
	6	3.66	1.00	-0.40	-0.38	12	3.17	1.23	-0.14	-0.94
Dominican Republic (*n* = 2,122)	1	3.74	0.99	-0.65	0.10	7	4.11	0.84	-0.86	0.72
	2	3.48	1.02	-0.30	-0.40	8	4.15	0.78	-0.75	0.62
	3	3.62	1.02	-0.43	-0.40	9	3.55	1.07	-0.41	-0.24
	4	3.53	1.03	-0.32	-0.53	10	3.12	1.09	-0.06	-0.52
	5	4.38	0.77	-1.30	1.90	11	3.23	1.13	-0.20	-0.62
	6	4.01	0.89	-0.68	0.09	12	3.50	1.15	-0.43	-0.52
Total sample (*n* = 18,528)	1	3.51	1.11	-0.49	-0.41	7	3.85	1.01	-0.76	0.17
	2	3.32	1.12	-0.24	-0.67	8	3.99	0.93	-0.88	0.67
	3	3.41	1.12	-0.32	-0.65	9	3.61	1.11	-0.50	-0.39
	4	3.33	1.13	-0.24	-0.75	10	3.26	1.16	-0.20	-0.69
	5	4.11	0.95	-1.08	0.90	11	3.32	1.16	-0.30	-0.68
	6	3.82	1.02	-0.68	-0.04	12	3.58	1.18	-0.55	-0.50

*Note. M=* Mean, *SD=* Standard deviation, *g1=* Skewness, *g2=* Kurtosis

**Fig. 1:**
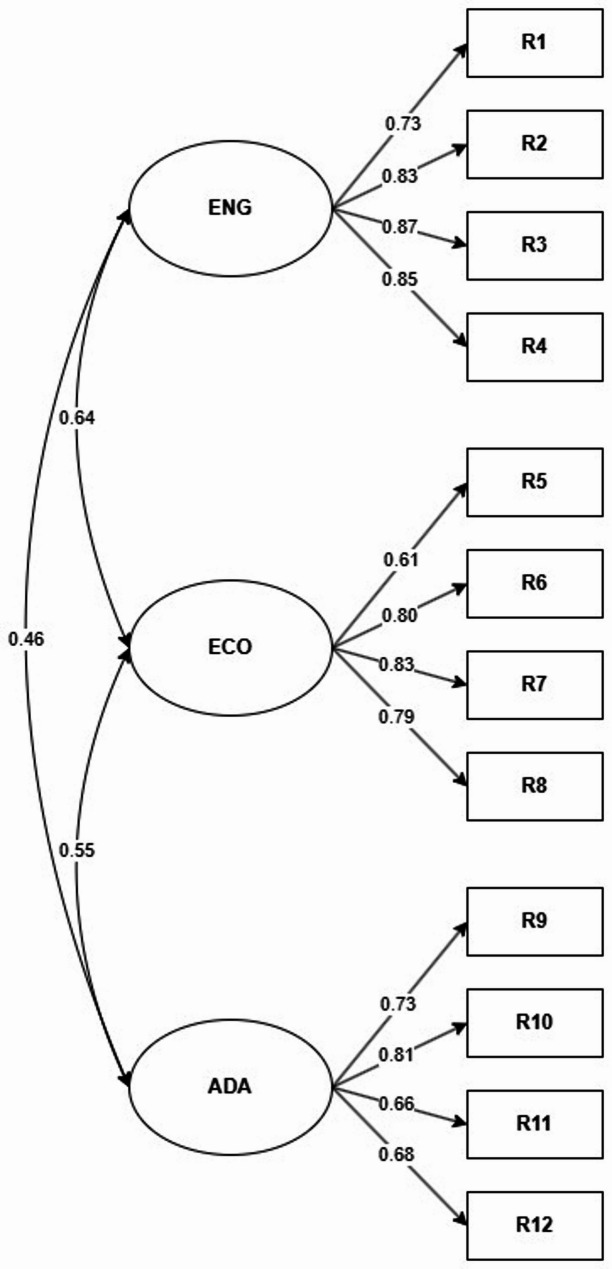
Three-factor correlated model of resilience in the total sample (*n* = 18,528)

Evidence of validity based on internal structure was demonstrated for all countries and for the total sample (Table 2), as satisfactory fit indices were obtained while retaining the three correlated factors with four consecutive items per dimension (Fig. 1). However, RMSEA values were slightly higher than expected in Ecuador, Panama, and Peru. Regarding reliability, it was satisfactory for the total sample and for most countries, except for El Salvador (ω < 0.70).

**Table 2:** Confirmatory fit indices and reliability by country and total sample, for the three-factor oblique model

Countries	X2 (gl)	*p*	CFI	TLI	RMSEA [IC 90%]	SRMR	ω		
							ENG	ECO	ADA
Chile (*n* = 2,385)	835.27 (51)	< 0.001	0.98	0.98	0.08 [0.08–0.09]	0.04	0.92	0.79	0.78
Colombia (*n* = 8,345)	2341.89 (51)	< 0.001	0.98	0.98	0.07 [0.07–0.08]	0.03	0.88	0.83	0.79
Ecuador (*n* = 1,822)	916.99 (51)	< 0.001	0.98	0.98	0.10 [0.09–0.10]	0.03	0.94	0.87	0.85
El Salvador (*n* = 1,956)	381.27 (51)	< 0.001	0.96	0.95	0.06 [0.05–0.06]	0.04	0.73	0.69	0.65
Mexico (*n* = 648)	286.10 (51)	< 0.001	0.98	0.97	0.08 [0.07–0.09]	0.05	0.91	0.81	0.81
Panama (*n* = 684)	336.77 (51)	< 0.001	0.96	0.95	0.09 [0.08–0.10]	0.05	0.90	0.78	0.79
Peru (*n* = 566)	267.63 (51)	< 0.001	0.98	0.97	0.09 [0.08–0.10]	0.05	0.92	0.79	0.81
Dominican Republic (*n* = 2,122)	802.39 (51)	< 0.001	0.97	0.96	0.08 [0.08–0.09]	0.05	0.90	0.76	0.75
Total Sample (*n* = 18,528)	4410.75(51)	< 0.001	0.98	0.98	0.07 [0.06–0.07]	0.03	0.88	0.80	0.79

*Note. ENG=* Engineering, *ECO=* Ecological, *ADA=* Adaptive

On the other hand, Table 3 shows that adequate ECV and PUC indices were not obtained, which suggests that the structure of the EEA cannot be considered bifactorial or as having a general dimension. In addition, the specific and general reliability coefficients were also below what was expected, especially in the ecological dimension in most countries.

**Table 3:** Confirmatory fit indices and reliability by country and total sample, for the bifactor model

Countries	X2 (gl)	*p*	CFI	TLI	RMSEA [IC 90%]	SRMR	ECV	PUC	ω			
									ENG	ECO	ADA	*R*-GEN
Chile (*n* = 2,385)	696.58 (42)	< 0.001	0.98	0.98	0.08 [0.08–0.09]	0.03	0.45	0.73	0.43	0.33	0.62	0.61
Colombia (*n* = 8,345)	1929.73 (42)	< 0.001	0.98	0.98	0.07 [0.07–0.08]	0.03	0.63	0.73	0.38	0.15	0.38	0.76
Ecuador (*n* = 1,822)	812.33 (42)	< 0.001	0.98	0.87	0.10 [0.09–0.10]	0.03	0.67	0.73	0.36	0.17	0.32	0.81
El Salvador (*n* = 1,956)	237.35 (42)	< 0.001	0.98	0.96	0.05 [0.04–0.06]	0.03	0.47	0.73	0.43	0.21	0.46	0.58
Mexico (*n* = 648)	207.23 (42)	< 0.001	0.98	0.97	0.08 [0.07–0.09]	0.04	0.50	0.73	0.40	0.25	0.61	0.67
Panama (*n* = 684)	227.22 (42)	< 0.001	0.98	0.97	0.08 [0.07–0.09]	0.04	0.45	0.73	0.36	0.39	0.63	0.61
Peru (*n* = 566)	189.63 (42)	< 0.001	0.99	0.98	0.08 [0.07–0.09]	0.03	0.41	0.73	0.51	0.38	0.63	0.59
Dominican Republic (*n* = 2,122)	664.74 (42)	< 0.001	0.98	0.96	0.08 [0.08–0.09]	0.04	0.48	0.73	0.42	0.29	0.54	0.63
Total Sample (*n* = 18,528)	3605.03 (42)	< 0.001	0.99	0.98	0.07 [0.06–0.07]	0.03	0.56	0.73	0.39	0.21	0.47	0.70

*Note. ENG=* Engineering, *ECO=* Ecological, *ADA=* Adaptive, *R-GEN=* General Resilience

Strict measurement invariance of the resilience instrument was demonstrated by sex and by country (Table 4), which allows for valid comparisons across groups (Svetina et al., 2019).

**Table 4:** Factorial invariance by country and sex

Variable	Level	X2 (gl)	*p*	CFI	ΔCFI	RMSEA	ΔRMSEA	SRMR
Country	Configural	5744.41 (408)	< 0.001	0.981		0.075		0.039
	Metric	5560.66 (471)	< 0.001	0.982	0.001	0.068	-0.007	0.042
	Scalar	5424.91 (450)	< 0.001	0.983	0.001	0.069	0.001	0.042
	Strict	5424.91 (450)	< 0.001	0.983	0	0.069	0	0.042
Sex	Configural	4565.81 (102)	< 0.001	0.981		0.069		0.033
	Metric	3925.39 (111)	< 0.001	0.983	0.002	0.061	-0.008	0.034
	Scalar	3870.49 (108)	< 0.001	0.984	0.001	0.061	0	0.034
	Strict	3870.49 (108)	< 0.001	0.984	0	0.061	0	0.034

Configural = unconstrained model; Metric = equal factor loadings; Scalar = equal intercepts; Strict = equal factor loadings, intercepts, and residual variances

Δ*CFI* Change in Comparative Fit Index, Δ*RMSEA* Change in Root Mean Square Error of Approximation

On the other hand, statistically significant differences by sex were found only in the engineering dimension, with women obtaining slightly higher mean scores. However, the effect size was trivial (*d* < 0.20; Domínguez-Lara, 2018). These results are detailed in Table 5.

**Table 5:** Differences in resilience by sex in the total sample (*n* = 18,528)

Variables	Sex	M (SD)	t	*p*	d	Magnitude
Engineering	Man	13.48 (3.73)	-4.13	< 0.001	-0.06	Trivial
	Woman	13.72 (3.78)				
Ecological	Man	15.77 (3.07)	-0.41	0.683	-0.01	Trivial
	Woman	15.79 (3.12)				
Adaptive	Man	13.73 (3.49)	-1.79	0.074	-0.03	Trivial
	Woman	13.83 (3.62)				

*Note. M=* Mean, *SD=* Standard deviation, *d=* Cohen’s d

On the other hand, in Table 6 the results showed statistically significant differences between countries in the three dimensions evaluated.

**Table 6:** Differences according to dimensions of resilience by country

Dimensions	Chile	Colombia	Ecuador	El Salvador	Mexico	Panama	Peru	Dominican Republic	F(7, 18520)	*p*	ω^2^
	M (SD)	M (SD)	M (SD)	M (SD)	M (SD)	M (SD)	M (SD)	M (SD)			
Engineering	13.32 (3.82)	13.77 (3.75)	13.20 (4.05)	12.84 (3.60)	12.83 (3.63)	13.65 (3.33)	13.31 (3.77)	14.37 (3.43)	36.09	< 0.001	0.01
Ecological	16.00 (2.81)	15.80 (3.13)	15.10 (3.42)	15.10 (3.33)	15.54 (3.04)	16.15 (2.81)	15.46 (2.97)	16.64 (2.50)	55.58	< 0.001	0.02
Adaptive	12.68 (3.65)	14.40 (3.37)	13.88 (3.75)	13.27 (3.50)	13.40 (3.55)	13.58 (3.46)	12.42 (3.85)	13.39 (3.33)	95.60	< 0.001	0.04

*Note. M=* Mean, *SD=* Standard deviation, ω^2^ = omega squared (effect size)

In the engineering resilience dimension, significant differences were observed between countries, with a small effect size. The mean values indicated that the Dominican Republic had the highest mean, followed by Colombia and Panama. In contrast, El Salvador and Mexico showed the lowest values in this dimension. Regarding ecological resilience, statistically significant differences were also identified between countries, with a small effect size. Again, the Dominican Republic obtained the highest mean, followed by Panama and Chile. Conversely, Ecuador and El Salvador presented the lowest means. Finally, in adaptive resilience, significant differences were found between countries, with a small effect size. In this dimension, Colombia recorded the highest mean, followed by Ecuador and Panama. In contrast, Peru and Chile showed the lowest means.

Table 7 presents the percentile-based classifications for resilience, distributed into five categories, where higher scores indicate a higher level of the construct.

**Table 7:** Classification percentiles for resilience (*n* = 18,528)

Percentile	Engineering	Ecological	Adaptive	Level
99	20	20	20	Very high
90	19	19	19	
80	17	18	17	High
70	16	17	16	
60	15	16	15	Average
50	14	15	14	
40	13	14	13	
30	12	13	12	Low
20	10	12	11	
10	9	11	10	Very low
5	7	10	8	

*Note.* Percentile ranks are based on the total sample (*n* = 18,528). Higher scores indicate higher levels of resilience. Classification levels follow the five-tier system used in previous EEA studies

Table 8 shows that for most countries, resilience levels in the engineering dimension are most frequently concentrated in the average category; however, in Ecuador and Mexico, the pattern differs, with low levels being more prominent. Regarding the ecological dimension, the average level predominates across all countries. Finally, in the adaptive dimension, all countries showed a greater proportion of average levels, except for Peru, where the very low level was predominant.

**Table 8:** Resilience levels in health sciences students by dimensions and countries

Engineering	Chile		Colombia		Ecuador		El Salvador		Mexico		Panama		Peru		Dominican Republic	
	*n*	%	*n*	%	*n*	%	*n*	%	*n*	%	*n*	%	*n*	%	*n*	%
Very high	329	13.8	1347	16.1	295	16.2	195	10	115	17.7	92	13.5	86	15.2	400	18.9
High	436	18.3	1649	19.8	341	18.7	291	14.9	90	13.9	111	16.2	87	15.4	457	21.5
Average	**623**	**26.1**	**2293**	**27.5**	307	16.8	**560**	**28.6**	178	27.5	**217**	**31.7**	**152**	**26.9**	**653**	**30.8**
Low	586	24.6	1923	23	**518**	**28.4**	555	28.4	**195**	**30.1**	191	27.9	151	26.7	412	19.4
Very low	411	17.2	1133	13.6	361	19.8	355	18.1	115	17.7	73	10.7	90	15.9	200	9.4
Ecological	n	%	n	%	n	%	n	%	n	%	n	%	n	%	n	%
Very high	496	20.8	1923	23	331	18.2	335	17.1	125	19.3	170	24.9	99	17.5	576	27.1
High	564	23.6	1541	18.5	280	15.4	385	19.7	106	16.4	164	24	117	20.7	536	25.3
Average	**922**	**38.7**	**3107**	**37.2**	**665**	**36.5**	**643**	**32.9**	**278**	**42.9**	**218**	**31.9**	**195**	**34.5**	**790**	**37.2**
Low	241	10.1	1011	12.1	309	17	285	14.6	77	11.9	103	15.1	108	19.1	160	7.5
Very low	162	6.8	763	9.1	237	13	308	15.7	62	9.6	29	4.2	47	8.3	60	2.8
Adaptive	n	%	n	%	n	%	n	%	n	%	n	%	n	%	n	%
Very high	223	9.4	1456	17.4	322	17.7	223	11.4	76	11.7	92	13.5	53	9.4	228	10.7
High	322	13.5	1965	23.5	345	18.9	301	15.4	108	16.7	108	15.8	66	11.7	339	16
Average	**738**	**30.9**	**2434**	**29.2**	**491**	**26.9**	**624**	**31.9**	**219**	**33.8**	**210**	**30.7**	169	29.9	**740**	**34.9**
Low	455	19.1	1540	18.5	353	19.4	379	19.4	118	18.2	156	22.8	101	17.8	443	20.9
Very low	647	27.1	950	11.4	311	17.1	429	21.9	127	19.6	118	17.3	**177**	**31.3**	372	17.5

*Note. *The values in bold indicate the level with the highest proportion

## Discussion

Health sciences students face a demanding educational pathway characterized by high academic workloads, long study hours, and complex emotional experiences. Together, these elements can exert a considerable influence on their psychological well-being and academic performance (Azim et al., 2025; Byrne et al., 2025). Under such circumstances, resilience becomes an important factor to develop, as it increases the likelihood that students will successfully cope with adverse events that arise not only within educational settings but also in social, family, and occupational contexts.

Given the relevance of this construct, it is essential to have instruments that measure resilience objectively, reliably, and validly among health sciences students, considering that adequate monitoring of its evolution may predict the quality of patient care they will provide in the future (Erschens et al., 2024; Wadi et al., 2024). To meet this need, the present study conducted a transcultural validation of the EEA Resilience Scale (Maltby et al., 2015) in Latin American health sciences students.

Initially, it was confirmed that, across all countries and in the total sample, the items were normally distributed at the univariate level, with skewness and kurtosis coefficients within acceptable ranges (± 2), a condition that supports the stability of subsequent estimations (Bandalos & Finney, 2019).

Regarding the structural functioning and reliability of the instrument, both were adequate across all contexts. This finding provides a solid methodological basis, suggesting that the underlying theoretical model remains robust at the transnational level. It is consistent with evidence showing that the three-factor resilience model (and not bifactor) has demonstrated structural stability across diverse populations worldwide (Maltby et al., 2016), and Latin America (Acosta-Martínez et al. 2024; Díaz-Narváez et al. 2025a, b).

Moreover, the EEA demonstrated strict measurement invariance across sex and country. Achieving this level of invariance indicates that factor loadings, item intercepts, and residual variances are equivalent across groups, suggesting that the measurement model operates in a statistically comparable manner across these populations. In practical terms, strict invariance supports the comparability of both latent means and observed scores, as it implies that group differences are unlikely to be attributable to measurement bias or differential item functioning (Svetina et al., 2019). This result strengthens the validity of cross-group comparisons, allowing more robust interpretation of potential differences in resilience profiles across countries and between men and women. Notably, these findings contrast with Maltby et al. (2016), who reported that achieving full invariance (particularly at the metric and scalar levels) can be challenging when applying the EEA model across different populations. Therefore, the present results provide additional support for the cross-cultural stability of the EEA framework within the studied sample.

Additionally, the study found that resilience levels in the engineering dimension tended to be slightly higher among women. This aligns with evidence suggesting that sex may influence resilience levels, often favoring women (Sull et al., 2015). However, it contradicts findings where men scored higher (Ammar et al., 2023; Mohammed et al., 2024; Zila-Velasque et al., 2024). It has been suggested that female students in health sciences tend to be more emotionally mature and sensitive than their male counterparts. They also demonstrate greater awareness of transitions and challenges they face, including how they adapt to change. This heightened emotional awareness and maturity may translate into a slightly greater capacity to modulate affective responses and to recover quickly after acute disruptions (Otaki et al., 2025).

Nonetheless, studies reporting no sex differences also exist (Al Omari et al., 2023; Chow et al., 2018). Therefore, it is important to emphasize that the association between sex and resilience is not homogeneous and may vary due to personal, contextual, and even genetic factors (Hu et al., 2015), reinforcing the notion that resilience is a dynamic and multifactorial construct.

Because percentile reference points offer a more practical approach for interpreting individual scores (Crawford & Garthwaite, 2009), five classification categories were proposed, consistent with previous studies involving health sciences students (Díaz-Narváez et al. 2025a). These norms may help identify students with relatively low or high resilience levels compared to their peers, contributing to diagnostic evaluation and the design of targeted interventions to strengthen coping capacity in the face of stressors inherent to medical training (Chye et al., 2024).

In this regard, the ecological dimension showed average levels in all countries, suggesting that students possess sufficient resources for system sustainability within the academic context (Maltby et al., 2017). That is, students generally exhibit tenacity, personal competence, and goal-directedness necessary to maintain academic progress despite the demanding nature of health sciences programs. However, the presence of low levels in the engineering dimension in Ecuador and Mexico may reflect the influence of demographic, cultural, economic, or social variables that negatively modulate this capacity. Indeed, transcultural comparisons have suggested that the meaning and average scores of resilience may vary across cultures (Maltby et al., 2016).

A more pronounced difference was observed in Peru, with very low levels in the adaptive dimension, suggesting a strong rigidity among students when adjusting to disruptions, which may increase their psychological vulnerability (Park et al., 2023), as adaptability is a central element in the resilience network (Maltby, 2025). Such a severe deficit may be particularly concerning for health sciences students in this country, who face constant challenges not only during clinical practice but also throughout their academic training (Azim et al., 2025).

### Study limitations

Despite these strengths, the present study must be interpreted in light of certain limitations. First, the use of non-probabilistic convenience sampling restricts the representativeness of the sample, limiting generalization to the entire population of health sciences students in the participating countries. Additionally, RMSEA fit indices were slightly higher than expected in Ecuador, Panama, and Peru, which may indicate cultural or sampling specificity influencing the measurement model. Reliability in El Salvador was lower than desired, suggesting the need for further studies to verify measurement consistency in that context.

Second, although overall model fit was satisfactory, the RMSEA values obtained in Ecuador, Panama, and Peru were slightly above commonly recommended thresholds. These deviations may reflect cultural, contextual, or sampling characteristics that warrant further examination. Similarly, internal consistency was lower than desirable in El Salvador, suggesting the need to investigate the functioning of specific items in that context.

Third, the cross-sectional design precludes conclusions about temporal stability or causal relationships. Future research should incorporate longitudinal and cross-lagged approaches to evaluate changes in resilience across the training years of health sciences students. Moreover, additional sources of validity (particularly convergent, discriminant, and predictive evidence) should be examined to complement the psychometric profile of the instrument.

Another consideration is the imbalance in sample sizes across sex and country groups in invariance testing, which could distort results, as larger groups exert more influence on model fit and can mask violations of cut-off criteria in smaller groups (Yoon & Lai, 2018). Future studies are encouraged to better control sample balance or apply statistical techniques to mitigate these implications. Although statistically significant sex differences were found in resilience scores, effect sizes were trivial. Applied interpretations should therefore acknowledge that these differences have limited practical relevance in educational, clinical, or social settings, and that other factors beyond sex may play a more prominent role in the development of resilience. Finally, additional evidence of validity—such as relations with other variables—should complement the psychometric analysis.

Finally, although country and sex differences were statistically significant in some dimensions, effect sizes were small. Therefore, the practical implications of these differences are limited, and future studies should explore alternative personal and contextual variables that may better explain variability in resilience among health sciences students across Latin America.

Despite these limitations, the transcultural validation of the EEA among health sciences students from eight Latin American countries underscores its practical relevance and supports its use as a psychometrically sound, reliable, and invariant instrument across country and sex groups. These findings ensure that scores are interpreted consistently across men and women as well as across countries, which is essential for multicenter research and for informing educational policies based on comparable data.

## Conclusions

The EEA demonstrated adequate psychometric properties, with a robust internal structure, good reliability, and evidence of factorial invariance by sex and country. The proposed percentile norms facilitate score interpretation and practical application within educational settings. Although significant country and sex differences were identified, their magnitude was trivial. However, one of the main strengths to highlight in the present study lies precisely there, as it is one of the few (if not the only) that provides comparisons of resilience at the level of Latin American countries specifically among health sciences students. Overall, the EEA emerges as a valid, reliable, and cross-culturally appropriate instrument for assessing resilience in Latin American health science students, although its reliability for the Ecological and Adaptive dimensions in El Salvador requires further research.

## Data Availability

The dataset supporting the conclusions of this article is available in the OSF repository: [https://osf.io/nprxt/overview? view_only=769fbe9249434f2c80e37ad1bf17c46d](https:/osf.io/nprxt/overview? view_only=769fbe9249434f2c80e37ad1bf17c46d).
